# ChiloDB: a genomic and transcriptome database for an important rice insect pest *Chilo suppressalis*

**DOI:** 10.1093/database/bau065

**Published:** 2014-07-04

**Authors:** Chuanlin Yin, Ying Liu, Jinding Liu, Huamei Xiao, Shuiqing Huang, Yongjun Lin, Zhaojun Han, Fei Li

**Affiliations:** ^1^Department of Entomology, College of Plant Protection, Nanjing Agricultural University, Jiangsu/The key laboratory of Monitoring and Management of Plant Diseases and Insects, Ministry of Agriculture, Nanjing, Jiangsu 210095, China, ^2^Department of Computer Science, College of Information Science and Technology, Nanjing Agricultural University, Nanjing, Jiangsu 210095, China and ^3^National Key Laboratory of Crop Genetic Improvement and National Centre of Plant Gene Research, Huazhong Agricultural University, Wuhan 430070, China

## Abstract

ChiloDB is an integrated resource that will be of use to the rice stem borer research community. The rice striped stem borer (SSB), *Chilo suppressalis* Walker, is a major rice pest that causes severe yield losses in most rice-producing countries. A draft genome of this insect is available. The aims of ChiloDB are (i) to store recently acquired genomic sequence and transcriptome data and integrate them with protein-coding genes, microRNAs, piwi-interacting RNAs (piRNAs) and RNA sequencing (RNA-Seq) data and (ii) to provide comprehensive search tools and downloadable data sets for comparative genomics and gene annotation of this important rice pest. ChiloDB contains the first version of the official SSB gene set, comprising 80 479 scaffolds and 10 221 annotated protein-coding genes. Additionally, 262 SSB microRNA genes predicted from a small RNA library, 82 639 piRNAs identified using the piRNApredictor software, 37 040 transcripts from a midgut transcriptome and 69 977 transcripts from a mixed sample have all been integrated into ChiloDB. ChiloDB was constructed using a data structure that is compatible with data resources, which will be incorporated into the database in the future. This resource will serve as a long-term and open-access database for research on the biology, evolution and pest control of SSB. To the best of our knowledge, ChiloDB is one of the first genomic and transcriptome database for rice insect pests.

**Database URL**: http://ento.njau.edu.cn/ChiloDB.

## Introduction

The rice striped stem borer (SSB), *Chilo suppressalis* Walker, is a serious rice pest that is distributed widely in the world. It damages rice from the seedling stage to maturity, causing huge yield losses. Different methods, including biological control, pheromone traps, planting resistance rice species and chemical insecticides, have been applied to control SSB. Among these control methods, chemical insecticides are the most widely used (1). However, chemical insecticides lead to considerable environmental pollution and represent a hazard to farmers and food safety (2). Thus, alternative strategies are needed to replace the use of chemical insecticides. The genomic sequence data will contribute dramatically to the biological interpretation and research on pest control.

*C.suppressalis* is a good model species to study insect adaptation to xenobiotic substances such as plant secondary metabolites, insecticides and other toxic chemicals. Insects eat plants as their main food resources and are the most successful herbivores. Plants have developed many successful strategies for defense against herbivores. One important strategy is to produce secondary metabolites that influence the behavior, growth or survival of herbivores. On the other hand, insect herbivores have evolved many ways to adapt to plant defense systems (3). SSB is a successful example of this. It is a polyphagous insect pest, and the SSB larvae feed on many kinds of plants, including rice, water bamboo, corn, sorghum, sugar cane, rape, broad bean, reed and barnyard grass. SSB may become an excellent model system to study morphogenesis development and insecticide resistance.

Several lepidopteran insect databases are publicly available, including SilkDB (4), KAIKObase (5), MonarchBase (6), Manduca Base (http://agripestbase.org/manduca/), Heliconius homepage (http://www.heliconius.org), DBM-DB (7) and KONAGAbase (8). These databases provide useful information systems for genomic analysis. Unfortunately, although rice is one of the most important food crops, no database for rice pests is currently available. We sequenced the genome of SSB using an Illumina sequencing platform, which generated whole genome shotgun (WGS) sequences that were assembled to obtain the first version of a draft genomic sequence. We also sequenced the SSB transcriptome. Here, we present ChiloDB, a web-accessible and species-specific resource that contains the genome and transcriptome sequencing data of *C.suppressalis*. This resource contains the scaffolds, coding sequences (CDS), microRNAs (miRNAs), piwi-interacting RNAs (piRNAs) and RNA Sequencing (RNA-Seq) data, which have been integrated with tools for genome annotation and comparative genomics analysis. ChiloDB has a user-friendly graphic user interface (GUI) that allows researchers to mine the data by sequence similarity and keyword searches. It also provides an information system for researchers to check gene annotation and submit information to the SSB genome-sequencing group (lifei03@tsinghua.org.cn).

## Data resources

The current data entries in ChiloDB are summarized in Table 1

**Table 1.** Summary of the data content of ChiloDB (9 December 2013)

Categories	Number
Scaffolds (length ≥2 Kb)	80 479
CDS	10 221
Proteins	10 221
MiRNAs	262
PiRNAs	82 639
Midgut transcriptome	37 040
Mixed sample transcriptome	69 977
Midgut downregulated genes	192
Midgut upregulated genes	21
Identified CYP genes or gene fragments	77

. It contains the gene information of SSB scaffolds, the first version of the official gene set (OGS) for SSB, the transcriptome, miRNA and piRNA. Ma *et al* sequenced the midgut transcriptome of *C.suppressalis* (9) from which we produced the genome scaffold, miRNA, piRNA and OGS data. All the data in ChiloDB are available at http://ento.njau.edu.cn/ChiloDB/. ChiloDB will be updated when the anticipated new version of the SSB genome and the gene annotations becomes available.

### Genome sequencing and *de novo* assembly

A WGS strategy was used to sequence the genome of *C.suppressalis*. Genomic DNA was extracted from 30 fifth-instar larvae using a DNeasy Blood and Tissue kit (Qiagen, Germany). The SSB larvae were collected from a single egg mass laid by a single female moth. About 5 µg DNA was sheared to fragments 170–800-bp long. The fragments were end-paired, A-tailed and ligated to sequencing adapters. The fragments were separated on agarose gels. They were then selected based on their sizes (190, 380, 500 and 700 bp) and amplified by ligation-mediated PCR to yield short insert size libraries (10). All the constructs were subjected to high-throughput sequencing using an Illumina HiSeq 2000 (CA, USA). The Illumina libraries were constructed and sequenced at the Beijing Genome Institute (BGI)-Shenzhen Co. Ltd. (Shenzhen, China). A total of ∼20.443 Gb (Giga base pairs) of raw data were obtained (Supplementary Table S1). The raw reads were cleaned by removing adaptor sequences, empty reads and low-quality reads using the Illumina software with default parameters. The ∼19.855 Gb of cleaned reads were used for assembly with the *de Bruijn* graph and SOAPdenovo software (11). The contig N50 was 5.2 kb and the GC content of the SSB genome was 31.27%. The genome was estimated by 17-mer analysis (12) to be ∼824 Mb in length. This WGS project has been deposited at DDBJ/EMBL/GenBank under accession number ANCD00000000. The version currently available in ChiloDB is the first version, ANCD01000000. The *C.suppressalis* genome-sequencing project has been submitted to GenBank (BioProject ID: PRJNA178139).

### OGS version 1

The OGS was obtained by annotating the SSB draft genome using the Optimized Maker-based Insect Genome Annotation (OMIGA) pipeline (13). After integrating the RNA-Seq data, we identified 10 010 protein-coding transcripts using the MAKER software (14). We also manually annotated 211 genes from the unassembled reads using exhaustive searches for odorant binding protein, chemosensory protein and cytoplasm P450 genes.

**Figure 1.**
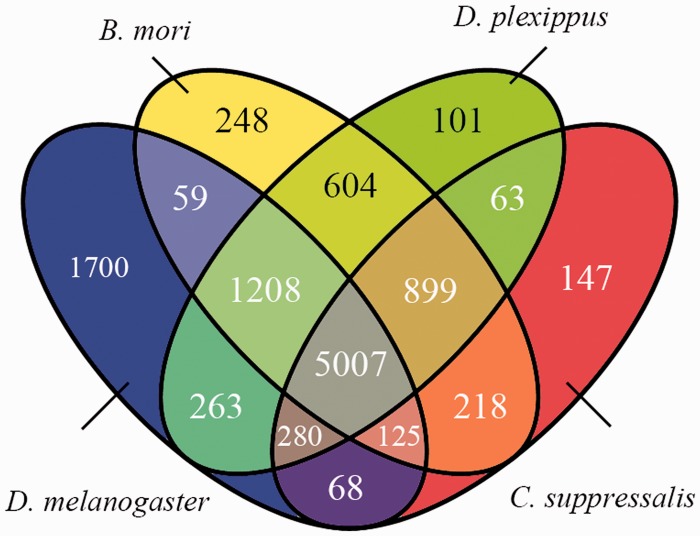
Venn diagram of the homologous protein-coding genes among four insects, *C.suppressalis*, *D.plexippus*, *B.mori* and *D.melanogaster*.

**Figure 2.**
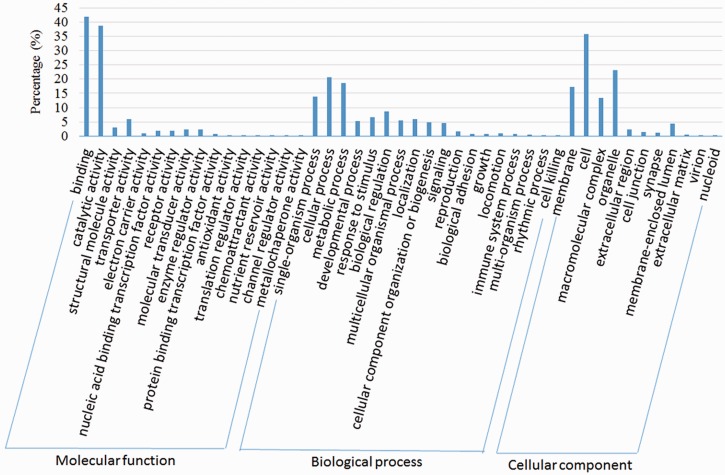
GO classification of the OGS in *C.suppressalis*.

**Figure 3.**
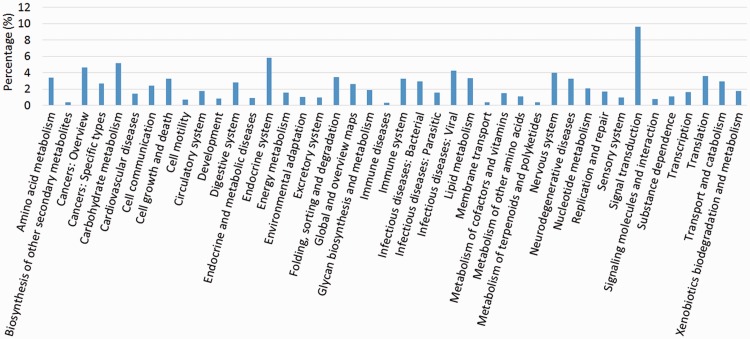
KEGG pathway analysis of the OGS in *C.suppressalis*.

We compared the predicted protein coding genes of *C.suppressalis* against the genomes of three other well-studied insects, *Drosophila melanogaster, Danaus plexippus* and *Bombyx mori*, using OrthoMCL-DB Version 5.0 (15) and identified 10 990 orthologs that corresponded to 10 221 *C.suppressalis* genes. *C.suppressalis* shared 218 and 68 orthologous genes with *B.mori* and *D.melanogaster*, respectively, while 147 genes were specific to *C.suppressalis.* A total of 5007 genes had orthologs in all four insects (Figure 1). We used the Blast2Go software (16) to annotate the protein-coding genes with Gene Ontology (GO) terms and found 27 286 GO terms associated with 5222 genes (Figure 2). A pathway analysis using the BLASTP software (17) to search the Kyoto Encyclopedia of Genes and Genomes (KEGG) database was also carried out (18) (Figure 3).

### Transcriptome *de novo* assembly

We sequenced the SSB transcriptome using an Illumina Genome Analyzer II (GA II) system. To generate as many gene transcripts as possible, we used a pooled sample of different developmental stages that included egg, larvae, pupa and adult. After removing the low-quality and contaminated reads, the remaining ∼6.8 Gb of data were assembled using Trinity with the default parameters (10). A total of 69 977 transcripts were obtained. The N50 of the mixed sample transcriptome was 596 bp. To annotate the transcriptome, the NCBI non-redundant (nr) database was searched using BLASTX (19) with a cutoff *E*-value of ≤1e-5. In total, 27 424 transcripts were annotated. The raw RNA-Seq data of the mixed sample transcriptome are available in the Sequence Read Archive (SRA) under accession number SRA060774.

We also downloaded the midgut transcriptome of SSB (SRA number SRA050703.2) from the NCBI SRA database. This transcriptome was sequenced from a midgut cDNA sample using the Illumina Genome Analyzer II (GA II) system. The data processing and statistics of the transcriptome have been described by Ma *et al.* (9). Briefly, after cleaning and quality checks, 39 million 90-bp-long reads were obtained. After assembly, 37 040 contigs were generated and the N50 was 576 bp. The mean transcript size was 497 bp, with lengths ranging from 201 to 9744 bp. Among the midgut transcripts, 15 446 showed significant similarity (*E*-value ≤1e-5) to known proteins in the nr database. After removing the redundant transcripts, 61 404 non-redundant transcripts remained. There were 31 948 transcripts that overlapped between the mixed sample and midgut transcriptomes.

### Repeat sequences

Repeat sequences were annotated from the SSB draft genome scaffolds using the RepeatMasker program version 3.0 with default parameters (20). Non-interspersed repeat sequences were found using the option ‘-noint’. Known transposable elements were identified by searching RepBase database (21), and high- and medium-copy repeat sequences were found using RepeatScout (22). Both class I (retroelements) and class II (DNA transposons) transposable elements were detected in the *C.suppressalis* genome (Table 2

**Table 2.** Classification of repeat sequences identified in the SSB genome

Repeat types	Number of elements*	Length occupied (bp)	Percentages of sequence (%)
Interspersed repeats			
SINE	17 429	1 807 922	0.26
LINE	45 118	6 198 632	0.9
LTR	24 705	2 600 951	0.38
DNA elements	48 832	6 115 414	0.88
Unclassified	2 436 483	230 557 335	33.29
Satellites	813	68 149	0.01
Simple repeats	39 357	1 695 156	0.25
Low complexity	333 986	10 507 620	1.53
Total base masked	2 946 723	259 551 179	38

). We found that 35.71% of the assembled SSB draft genomes were interspersed repeats.

### MiRNA and piRNA

MiRNA genes were identified from a small RNA library using miRDeep (23). The scaffolds were used as the reference SSB genome. In total, 262 miRNAs were collected for SSB, among which 217 miRNAs were highly conserved in metazoans and 45 were novel. The piRNA genes were predicted from the same small RNA library using piRNApredictor (24).

### Genes coding for cytochrome P450

The cytochrome P450 (CYP) superfamily is a large group of proteins involved in various physiological processes. In insects, CYPs participate in the synthesis and degradation of many physiologically important compounds such as ecdysteroids, juvenile hormones and pheromones. A total of 77 CYP genes have been discovered in the SSB genome and transcriptome by sequence analyses (25). RT-PCR amplification confirmed the validity of these CYP genes. Among these CYP genes, 28 were reported to have intact open reading frames. The nomenclature and classification of CYPs are based on similarities among the amino acid sequences of the proteins that they encode. For example, CYP proteins with sequence identities >40% belong to a family, and CYPs with 55% identities belong to a subfamily. When the SSB CYPs were classified into four clans (mitochondrial, CYP2, CYP3 and CYP4), we found that there had been an apparent expansion of the CYP3 clan because nine members of the CYP6AB subfamily of the CYP3 clan were detected in the SSB genome. The phylogenetic tree of the CYP genes is available from the download pages in ChiloDB.

## Database construction

### Database system implementation

**Figure 4.**
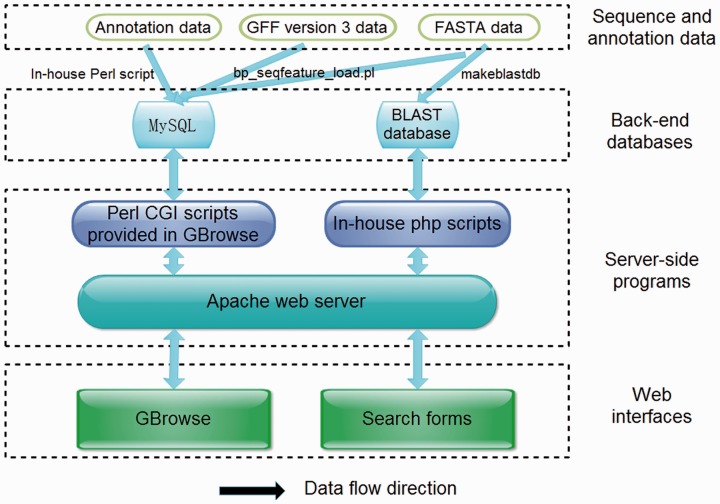
Overview of the ChiloDB architecture.

ChiloDB was developed on an Apache HTTP server in a Linux (Redhat 5.6) operating system. The web pages were written using PHP, html language, Cascading Style Sheets (CSS) and JavaScript. Custom Perl scripts were used to make the database user-friendly with a good interaction interface. The Apache server handles queries from web clients through PHP scripts to perform searches. The generic Genome Browser (GBrowse 2.0) package, a component of the Generic Model Organism Project (26, 27), was used for genome visualization. The tool allows researchers to obtain gene structure information. A local Basic Local Alignment Search Tool (BLAST) server has also been installed in the ChiloDB system. An overview of the ChiloDB architecture is given in Figure 4.

### BLAST server

In the ChiloDB system, we used the wwwblast program version 2.2.26 (28) to implement BLAST sequence similarity searches against the SSB genome and transcriptome sequences because it provides a GUI through the web forms. The makeblastdb program of the stand-alone NCBI BLAST 2.2.28+ software package (29) was used to create the BLAST alignment database. In the ChiloDB system, users can search against the different kinds of SSB sequence data, including scaffolds, CDSs and transcriptomes. All the sequences are well annotated, which will contribute to SSB genomic research (Figure 4).

### Gene search

ChiloDB allows users to search for gene information of interest using keywords, GeneID (from CSUOGS100001 to CSUOGS110221), GO ID or GO term and KEGG ID or KEGG annotation. Annotation keywords and gene names can also be used to retrieve gene information from ChiloDB. The search results provide related gene sequences and their annotations.

### Genome visualization

GBrowse is a well-known browser that integrates database and interactive web pages to display genome annotations (30). We used GBrowse in ChiloDB to provide interactive views of genome information and to navigate the annotations along with the genome scaffolds. The genome data (in FASTA format) along with the GFF (General Feature Format version 3) data required for the GBrowse are stored in a MySQL database using bp_seqfeature_load.pl provided by BioPerl. The ChiloDB GBrowse provides a tracking function for CDS, exon, repeat sequences, DNA/GC content, miRNA, piRNA, coverage of the transcriptome reads and the CDS structure of homologous genes in SilkDB (4) and FlyBase (31). Pop-up balloons in the gene model track display links to gene sequences of interest.

### Download page

FTP and HTTP links are provided for users to download the entire data sets. The ChiloDB FTP site (ftp://chilodb.in sect-genome.com/pub/) contains genomic scaffolds (draft genome version 1), predicted OGS (version 1) in FASTA format, the genomic position of repeat sequences, gene structure in GFF3 format, predicted miRNA and piRNA genes and the sequences of the CYP gene family. Gene annotations include gene functional descriptions as well as KEGG and GO annotations. The SSB midgut transcriptome data and the pooled mixed sample data in FASTA format are also available in ChiloDB.

## Conclusion

**Figure 5.**
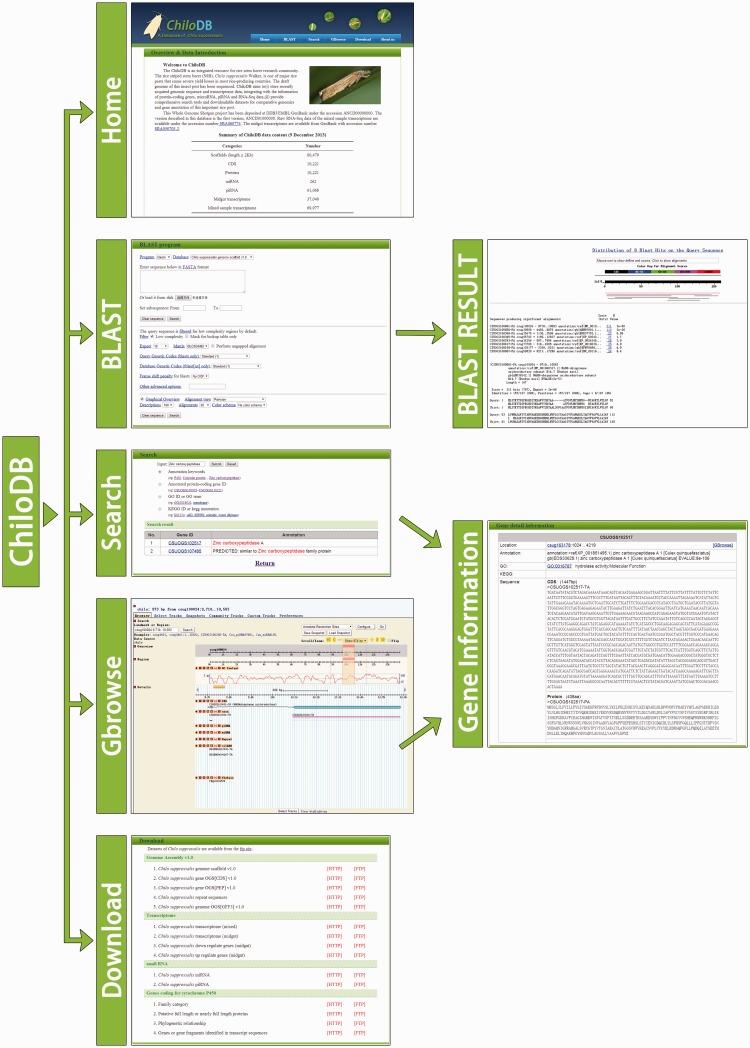
Organizational structure of the ChiloDB web pages.

We have developed ChiloDB, a genomic and transcriptome database for the SSB *C.suppressalis*. ChiloDB provides comprehensive and varied information for protein-coding genes, non-coding genes (miRNA and piRNA) and the draft genome sequences of SSB. To the best of our knowledge, this is the first database for a rice insect pest, and we expect that it will make a substantial contribution to the genome sequencing initiative of the i5k Insect and other Arthropod Genome Sequencing Initiative (http://www.arthropodgenomes.org/wiki/i5K). ChiloDB has user-friendly GUI-based web interfaces (Figure 5) that allow users to search and acquire gene sequences of interest easily and efficiently. Currently, we are carrying out another large-scale sequencing of the SSB genome to improve the annotation quality, so that many more important gene families and pathways can be identified. When the new version of the genomic data becomes available, ChiloDB will be updated. In the future, we aim to develop ChiloDB as a comprehensive information system for SSB researchers (and the whole insect stem borer research community) by integrating Chilo People (researchers who study SSB or rice stem borers), Chilo Publications (published papers on SSB or rice stem borers) and Chilo pest control (strategies used to control SSB and rice stem borer) into the database.

## Supplementary Data

Supplementary data are available at *Database* Online.

## Funding

The National High Technology Research and Development Program (“863”Program) of China [grant number 2012AA101505]; the National Science Foundation of China [grant number 31171843, 31301691]; and the Jiangsu Science Foundation for Distinguished Young Scholars [grant number BK2012028].

*Conflict of interest*. None declared.
